# Beyond the usual – Atypical imaging presentation in lung cancer and implications for TNM-staging

**DOI:** 10.1097/CCO.0000000000001203

**Published:** 2025-11-07

**Authors:** Daria Kifjak, Rebecca Mura, Svitlana Pochepnia, Nino Bogveradze, Aida Korajac, Benedikt H. Heidinger, Ruxandra-Iulia Milos, Lucian Beer, Helmut Prosch

**Affiliations:** a Department of Biomedical Imaging and Image-guided Therapy; b Christian Doppler Laboratory for Machine Learning Driven Precision Imaging, Department of Biomedical Imaging and Image-guided Therapy, Medical University of Vienna, Vienna, Austria; c American Hospital Tbilisi, Tbilisi, Georgia; d Department of Diagnostic and Interventional Radiology, Clinic Donaustadt, Vienna Healthcare Group, Vienna, Austria

**Keywords:** computed tomography, lung cancer, TNM staging

## Abstract

**Purpose of review:**

This review illustrates the spectrum of atypical computed tomography (CT) appearances of lung cancer and provides practical guidance for accurate diagnosis and staging.

**Recent findings:**

While most lung cancers show typical CT features, certain subtypes present atypically, mimicking benign conditions or other malignancies. These include pneumonic-type adenocarcinoma, multifocal adenocarcinoma with ground-glass/lepidic features, lung cancer with air lucency, and pulmonary carcinoid tumors. Pneumonic-type adenocarcinoma often resembles infectious pneumonia, requiring careful CT evaluation, and tissue sampling for confirmation. Multifocal ground-glass/lepidic adenocarcinomas, most commonly seen in female never-smokers, are indolent with low metastatic potential. Lung cancers with air lucency, appearing as cysts, cavitary, or bullous lesions, challenge volumetric assessment and may benefit from adapted TNM measurements excluding air-space components. Pulmonary carcinoid tumors show variable imaging features and require tailored staging and management based on their differentiation and spread.

**Summary:**

Accurate recognition of atypical CT manifestations of lung cancer is critical to avoid misinterpretation and inappropriate management. Integrating imaging characteristics with histopathologic and, when applicable, molecular data ensures correct staging and guides personalized therapy.

## INTRODUCTION

Lung cancer remains the second most common malignancy and the leading cause of cancer-related deaths worldwide in 2025, accounting for a substantial global health burden despite advances in screening, diagnostic imaging, and targeted therapies [1^▪▪^,^2^]. Accurate staging is crucial, as it determines both prognosis and therapeutic strategies. The TNM (tumor, nodule, and metastasis) classification is used internationally as a standard framework in lung cancer staging, with imaging, particularly computed tomography (CT), playing a central role in initial diagnosis, lesion characterization, and staging [3^▪▪^,4^▪^].

While many lung cancers present with typical CT features that facilitate a straightforward diagnosis and staging, a subset of them may show atypical imaging appearances that may deviate from the classic presentation. These atypical manifestations can overlap with benign pulmonary conditions (e.g., infections), as well as with other malignant processes, leading to potential misinterpretation, delayed diagnosis, and staging inaccuracies [5].

Lung cancer typically presents as a solitary nodule (<3 cm) or a mass (≥3 cm) within the lung parenchyma. Both nodules and masses may show variable shapes, including round, ovoid or polygonal, with margins ranging from well defined to irregular (e.g., lobulated and spiculated). Whereas nodules may be solid, part-solid or purely ground-glass nodules, masses usually show solid soft tissue attenuation. However, lung carcinomas may also exhibit cavitary, cystic, or calcified components [6^▪▪^]. Additionally, the lesion location often reflects the histologic subtype: in fact, adenocarcinomas tend to occur peripherally, while squamous cell carcinomas are more commonly centrally located [5].

An atypical presentation of lung cancers encompasses a broad spectrum of distinct entities. These include multifocal purely ground-glass opacities suggestive of adenocarcinoma *in situ* (AIS) or minimally invasive adenocarcinoma (MIA); pneumonic-type adenocarcinoma, particularly mucinous subtypes, which can mimic infectious pneumonia; and lung cancers arising within preexisting cystic spaces [7–9]. Finally, lung cancers may appear as endobronchial masses, including typical and atypical carcinoids [10].

Given the heterogeneity of imaging appearances, these atypical forms pose unique diagnostic and staging challenges. Misclassification may alter stage assignment and lead to inappropriate treatment choices. Therefore, radiologists must be familiar with the spectrum of atypical CT features, their histopathologic correlates, and the nuances of staging according to the TNM system. Staging imaging modalities should ideally include PET/CT and cranial MRI; however, if these are not available, staging can rely on CT scans and Tc-99m bone scintigraphy alone [11]. This review aims to provide a comprehensive overview of atypical CT presentations of lung cancer and offer practical guidance to support accurate diagnosis and staging in these complex scenarios. 

**Box 1:**
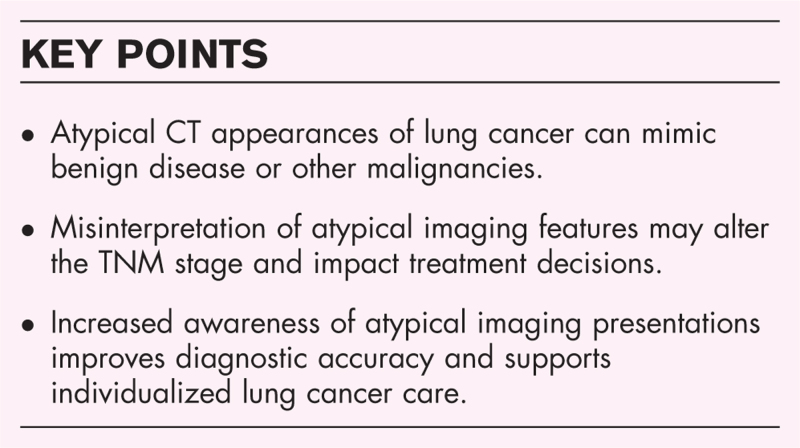
no caption available

## LUNG CANCER WITH ATYPICAL PRESENTATION

### Pneumonic-type adenocarcinoma

**FIGURE 1:**
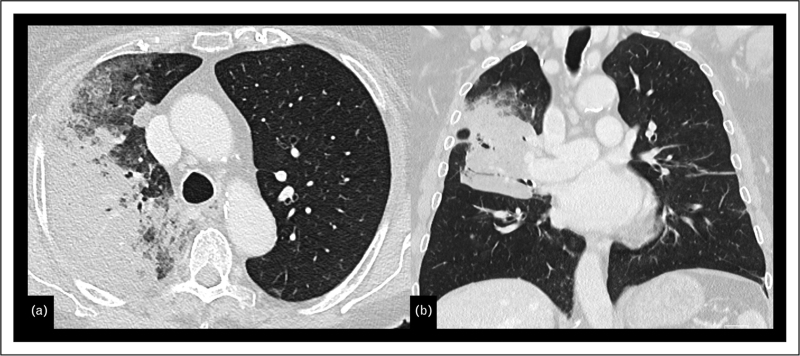
Transverse (a) and coronal (b) CT image reconstructions of the lung show a consolidation with air-bronchogram in the right upper and middle lobes. As the tumor is located in two different ipsilateral lobes and due to the tumor size, it is categorized as T4.

Pneumonic-type adenocarcinoma refers to an atypical form of lung cancer that mimics pneumonia on imaging, and, histologically, most often, but not exclusively, corresponds to invasive mucinous adenocarcinoma [7]. CT is the imaging modality of choice for the evaluation of patients with a suspected pneumonic type adenocarcinoma. The CT characteristic features include consolidations with air-bronchograms and ground-glass opacities, typically with a peribronchial distribution (Fig. 1) [7,12]. According to Jung *et al.*[13], CT findings of air-filled bronchus, stretching/squeezing/widening of the branching angle, the angiogram sign and bulging of the fissure, may help differentiate pneumonic-type adenocarcinoma from infectious processes [14,15]. The pathologic substrate of this pneumonic component involves partial or complete filling of alveolar airspaces by mucin or tumor cells as well as by inflammatory cell infiltration and fibrotic stromal reaction [8].

The diagnostic role of ^18^Fluordeoxyglucose(FDG)-PET/CT is limited and can result in false-negative findings and reports. Indeed, even though invasive mucinous adenocarcinomas may exhibit SUVmax values higher than those observed in infectious pneumonias, lesions with predominant lepidic growth often show little or even no FDG avidity [16,17]. Therefore, a definitive diagnosis usually requires pathological confirmation through invasive procedures such as transthoracic biopsy, bronchoscopy with bronchoalveolar lavage (BAL), or transbronchial biopsy.

Although patients with pneumonic-type adenocarcinomas often present with diffuse pulmonary involvement, nodal or extrathoracic metastases are infrequently observed [7]. From a staging perspective, when a single lesion is present, the T category is determined by its size and/or the invasion of the adjacent structures, whereas in case of multiple consolidations, the staging depend on their location: T3 if confined to one lobe, T4 if involving different ipsilateral lobes, or M1a if involving both lungs [3^▪▪^].

### Multifocal lung adenocarcinoma with ground-glass/lepidic features

Multifocal lung adenocarcinomas with ground-glass/lepidic (GG/L) features typically correspond to various subtypes of adenocarcinoma, including minimally invasive adenocarcinoma (MIA), adenocarcinoma *in situ* (AIS), primarily lepidic-predominant adenocarcinoma (LPA), and atypical adenomatous hyperplasia (AAH), with ground-glass and solid components reflecting lepidic and invasive histologic patterns, respectively [7]. This entity, typically seen in female never-smokers, is regarded as distinct and indolent with a favorable prognosis, a low metastatic potential (0–15% of distant metastases), and a tendency to develop additional subsolid lesions [7,18^▪^,^19^,^20^].

**FIGURE 2:**
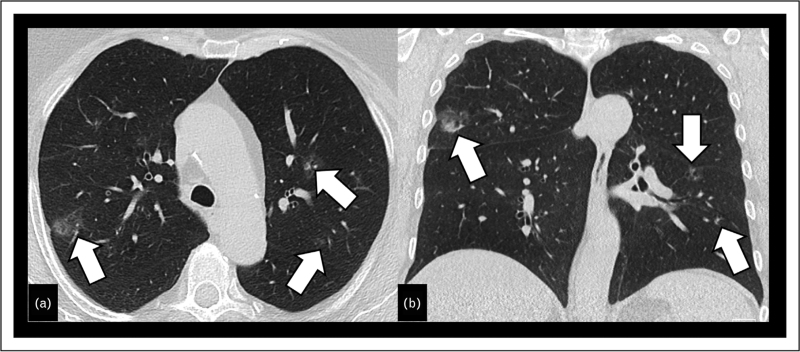
Transverse (a) and coronal (b) CT image reconstructions of the lung show multiple sub-solid nodules in both lungs. The largest lesion, located in the right upper lobe, measures 1.8 cm in total with a solid component measuring 0.6 cm (best appreciated on coronal reconstruction); there are three lesions (arrows), making it a T1a (3/m).

Multifocal lung adenocarcinoma with GG/L features radiologically presents as multiple sub-solid nodules, either pure ground-glass or part-solid (Fig. 2) [7,21,22]. Advances in imaging have markedly increased the detection of such lesions, highlighting the importance for radiologists to recognize that multiple lung nodules do not necessarily mean metastases or advanced-stage disease. In fact, numerous studies show that they often represent synchronous primary tumors rather than metastatic disease. Histopathologic, immunophenotypic, and sometimes molecular features can aid in differentiating primary lung cancers from metastatic lesions, an essential step, as the relationship between two nodules fundamentally guides the appropriate treatment approach [18^▪^,^19^].

Multifocal lung adenocarcinomas with GG/L features are staged according to the current TNM classification, in which the T category is based on the size of the solid (invasive) component of the largest nodule, with the number of nodules (#) or (m) to indicate multiplicity, while N and M categories are assigned collectively [3^▪▪^].

Importantly, in clinical practice, when patients present with multiple nodules, a critical distinction must be made between intrapulmonary metastases and synchronous primary lung cancers, as this has major implications for staging and treatment [3^▪▪^,^21^,^23^,^24^]. Intrapulmonary metastases are usually characterized by separate nodules that share the same morphology and histologic subtypes as the primary tumor, often accompanied by nodal or distant metastases [23]. Staging of intrapulmonary metastases is determined by the location of the additional nodule(s): T3 if the metastasis is located in the same lobe as the primary tumor, T4 if in a different ipsilateral lobe, and M1a if in the contralateral lung, with a single N and M category assigned [3^▪▪^,^24^,^25^]. Morphology, histology, and nodal status remain the key criteria to discriminate between intrapulmonary metastases and multifocal primary lung cancer [23].

Synchronous primary lung cancers represent two or more distinct and unrelated tumors, frequently showing different histological types with a different growth rate, different imaging morphologies and metabolic activity, typically lacking nodal metastases [23,26^▪▪^,^27^]. In such cases, each tumor must be staged separately with its own c-TNM and p-TNM classification [3^▪▪^].

### Lung cancer with air lucency

In up to 18% non-small cell lung cancer (NSCLC), especially in high-risk patients, can present as an air lucency, the majority of which are adenocarcinomas. The term lung cancer with air-lucency includes lesions which present as either cysts, cavities, pseudo-cavities, emphysematous bullae or have a bubble-like appearance. They are typically slow growing and relatively frequently missed [9]. To highlight the importance of cystic lesions, the American College of Radiology (ACR) included the subcategory of “atypical pulmonary cyst” in the current 2022 version of the Lung CT Screening Reporting and Data System (Lung-RADS) [28^▪^].

**FIGURE 3:**
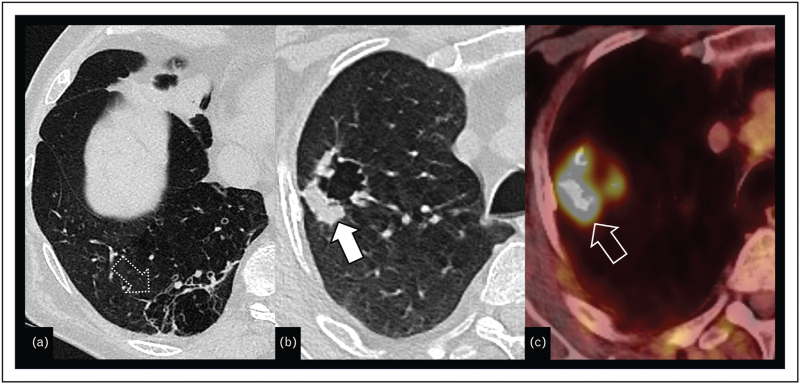
Transverse CT image reconstruction of the right lung (a) shows a thin-walled cyst (dashed arrow), without any measurable soft-tissue component. Transverse reconstruction of CT (b) and PET/CT (c) images show a cystic lesion with an asymmetric wall-thickening and a distinct soft-tissue component (white arrow), which shows an FDG-uptake (open arrow).

According to Mascalchi *et al.*[29] and Maki *et al.*[30], cystic lesions on CT may appear with a focal, exophytic (type 1) or endophytic (type 2) solid nodule, circumferential or asymmetrical wall thickening (type 3) or as a multilocular cystic air-space with interposed solid tissue (type 4) (Fig. 3) [31]. Although there are no distinct CT patterns which reliably differentiate between malignant and benign features of cystic lesions, CT is the standard imaging method for follow-up and monitoring [31]. An increase in size of the solid component, an increasing solidification or a missing response to antiinfectious treatment are signs of malignancy [9,31,32]. Despite its distinctive morphology, lung cancer with air lucency is staged according to the current TNM classification. Volumetric assessment can be difficult if a lesion contains a cystic or nonsolid component, and its exophytic or endophytic components are comparatively small. In lesions having one focal exophytic or endophytic component, a large proportion of the lesion consists of air, thus measuring the entire lesion diameter may lead to an overestimation of the tumor burden [9,33]. Based on the assumption that the solid portion of a lesion corresponds to its invasive component, it has been proposed, similar to the TNM staging of subsolid nodules, that only the solid part should be measured and staged, thereby excluding the tumor's air-space component [9,33–35]. If a focal solid nodule is absent, it is recommended to measure the maximal wall thickness [9]. Although ^18^FDG-PET/CT plays an important role in lung cancer staging, its role in staging lung cancer with air-lucency was shown to be ambiguous [9]. Indeed, although it has proven useful in lesions with a solid component more than 8 mm, its utility in thin-walled cystic lesions is rather limited, with no FDG-uptake observed in 83% of the cases [9,36,37]. Furthermore, one has to take into account that this entity shows a relatively high rate (about 30%) of synchronous or prior lung cancers [9].

### Lung carcinoid tumors

Carcinoid tumors of the lung are rare neuroendocrine epithelial tumors, which range from well differentiated, low-grade, less aggressive, typical carcinoids to poorly differentiated, intermediate-grade, more aggressive atypical carcinoids [10]. Most carcinoids are small, slow-growing and centrally located (80%), whereas approximately 20% arise in the lung periphery [10,38]. The diagnosis is usually established in the early stages. In advanced stages, common metastatic sites include the liver and bones in case of typical carcinoids, whereas atypical carcinoids usually metastasize also to soft tissue, brain, spleen and adrenal glands. Although biomarkers including chromogranin A, synaptophysin, CD56 and TTF-1 are used to confirm the diagnosis, imaging plays a pivotal role in determining the extent of the disease, thereby guiding staging [10]. Both typical and atypical carcinoids are recommended to be staged according to the TNM classification of lung cancer. However, the overlap between combined stages and subcategories within the staging system reduces the precision of the TNM classification, especially in the intermediate stages [39].

Contrast-enhanced CT was proven to be sensitive in detecting lymph nodes, tumorlets, diffuse idiopathic pulmonary neuroendocrine cell hyperplasia (DIPNECH) and in evaluating tumor margins [38,40]. In most cases, CT imaging features are nonspecific and can resemble those of adenocarcinoma or squamous cell carcinoma. Peripherally located lesions include round or ovoid-shaped nodules with smooth or lobular margins and contrast medium enhancement. Centrally located lesions frequently reveal indirect signs, including bronchial obstruction leading to atelectasis, obstructive pneumonia, air trapping or, less commonly, bronchiectasis or lung abscesses. In suspected DIPNECH, additional high-resolution CT (HRCT) with expiratory scans showing mosaic attenuation and air-trapping might be useful [40]. Although MRI shows less sensitivity in detecting small lesions, it could be helpful in localizing liver and bone metastases [10,38,40]. Somatostatin receptor scintigraphy was shown to detect about 70% of carcinoids, but

**FIGURE 4:**
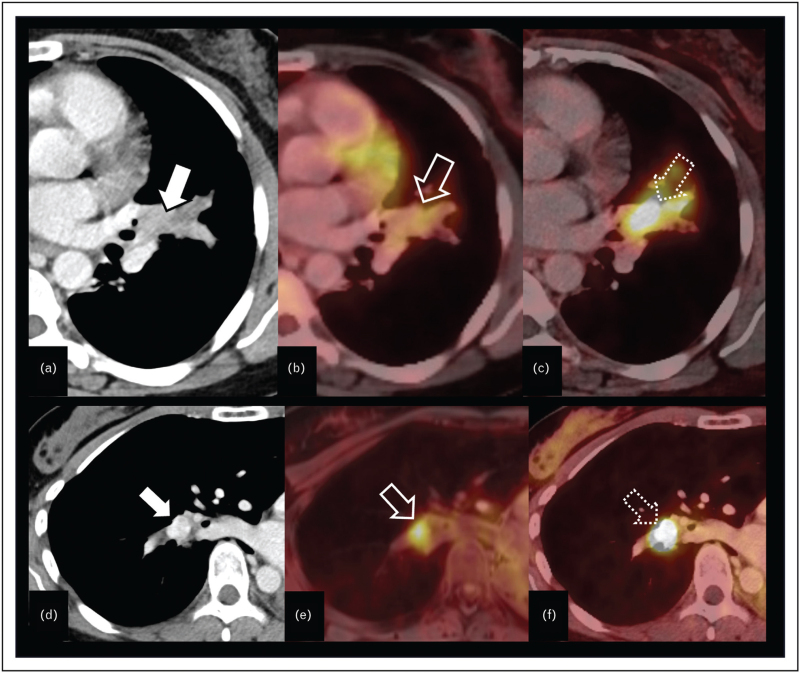
Transverse CT image reconstruction shows an endobronchial tumor (a) (white arrow) on the left, which does not show any FDG-uptake (open arrow) on ^18^FDG-PET/CT scan (b) but somatostatin expression (dashed arrow), and therefore positivity on DOTANOC-PET CT scan (c), highly suggestive of a typical carcinoid. Transverse CT image reconstruction shows an endobronchial tumor (d) (white arrow) on the right, which shows an FDG-uptake (open arrow) on ^18^FDG-PET/CT scan (e) and somatostatin expression (dashed arrow) and therefore positivity on DOTANOC-PET CT scan (f), highly suggestive of an atypical carcinoid.

PET/CT with ^68^Ga-DOTANOC, ^68^Ga-DOTATOC, or ^68^Ga-DOTATATE shows a higher sensitivity for tumors expressing somatostatin receptors, making it suitable for preoperative staging as well as for postoperative surveillance [38,41,42] (Fig. 4a-c). ^18^FDG-PET/CT was also shown to be valuable for detecting lymph node and distant metastases in atypical carcinoids, which, due to a higher proliferation index, have a higher rate of distant metastases (Fig. 4d-f). In typical carcinoids however, the sensitivity of ^18^FDG-PET/CT for the detection of nodal metastases is rather low (33%) as compared to atypical carcinoids (94%); thus, false-negative results are common, making ^18^FDG-PET/CT the most sensitive imaging technique for atypical carcinoids [40,43,44]. When treatment decisions rely on the N2 status, additional invasive mediastinal staging involving endobronchial or endoscopic ultrasound fine needle aspiration, or alternatively mediastinoscopy is recommended [40].

## LIMITS AND FUTURE PERSPECTIVES

A major limitation in the assessment of atypical lung cancer presentations lies in the overlap of imaging features with benign disease and other malignancies, which can lead to misclassification and inappropriate staging. Current TNM staging criteria, which are standardized, may not fully account for unique morphological patterns such as cystic airspaces, potentially impacting prognostic accuracy. In addition, variability in the use and interpretation of advanced imaging modalities, including PET/CT and MRI, limits cross-center consistency. Future research should focus on refining systems for atypical morphologies, integrating molecular results, volumetric and texture-based metrics, as well as artificial intelligence for improved lesion characterization. Multicenter prospective studies combining imaging, histopathology, and molecular profiling will be essential to develop evidence-based guidelines for these complex cases.

## CONCLUSION

Atypical presentations of lung cancer on CT pose significant diagnostic and staging challenges, with implications for prognosis and treatment selection. Recognizing entities such as pneumonic-type adenocarcinoma, multifocal adenocarcinoma with ground-glass/lepidic features, lung cancer with air lucency, and pulmonary carcinoid tumors is essential to avoid misinterpretation and inappropriate management. Detailed assessment of imaging features, correlation with histopathologic and, when appropriate, molecular findings, together with careful application of the TNM staging principles, is key to accurate classification. Ultimately, heightened awareness of these atypical patterns enables radiologists to contribute more effectively to precise diagnosis and individualized patient care.

## Acknowledgements

*The financial support by the Austrian Federal Ministry of Labor and Economy, the National Foundation for Research, Technology and Development, and the Christian Doppler Research Association is gratefully acknowledged.*

### Financial support and sponsorship

*None.*

### Conflicts of interest

*Beer: Speaker honoria: AstraZeneca, MSD, Novartis, Roche, Bayer; Prosch: Speaker honoria: AstraZeneca, BMS, Boehringer Ingelheim, Janssen, MSD, Novartis, Roche, Sanofi, Siemens Healthcare, Takeda. Advisory Boards: BMS, Boehringer Ingelheim, MSD, Roche/Intermune, Sanofi. Travel grants: Boehringer Ingelheim. Research support: Boehringer Ingelheim, AstraZeneca, Siemens Healthineers, Austrian Federal Ministry for Labour and Economy, the National Foundation for Research, Technology and Development and the Christian Doppler Research Association, EU Commission (EU4Health, Horizon Europe Health). The other authors declare no conflict of interest.*

## REFERENCES AND RECOMMENDED READING

Papers of particular interest, published within the annual period of review, have been highlighted as:

▪ of special interest
▪▪ of outstanding interest
